# The Nrd1–Nab3–Sen1 transcription termination complex from a structural perspective

**DOI:** 10.1042/BST20221418

**Published:** 2023-05-24

**Authors:** Belén Chaves-Arquero, José Manuel Pérez-Cañadillas

**Affiliations:** 1 Department of Structural and Chemical Biology, Center for Biological Research ‘Margarita Salas’, CIB, CSIC, Av. Ramiro de Maeztu 9, 28040 Madrid, Spain; 2 Department of Biological Physical Chemistry, Institute of Physical-Chemistry ‘Blas Cabrera’, CSIC, C/Serrano 119, 28006 Madrid, Spain

**Keywords:** CUTs, Nab3, Nrd1, pervasive transcription, protein structure, Sen1

## Abstract

A substantial part of living cells activity involves transcription regulation. The RNA polymerases responsible for this job need to know ‘where/when' to start and stop in the genome, answers that may change throughout life and upon external stimuli. In *Saccharomyces cerevisiae,* RNA Pol II transcription termination can follow two different routes: the poly(A)-dependent one used for most of the mRNAs and the Nrd1/Nab3/Sen1 (NNS) pathway for non-coding RNAs (ncRNA). The NNS targets include snoRNAs and cryptic unstable transcripts (CUTs) generated by pervasive transcription. This review recapitulates the state of the art in structural biology and biophysics of the Nrd1, Nab3 and Sen1 components of the NNS complex, with special attention to their domain structures and interactions with peptide and RNA motifs, and their heterodimerization. This structural information is put into the context of the NNS termination mechanism together with possible prospects for evolution in the field.

## Introduction

**Figure 1.**
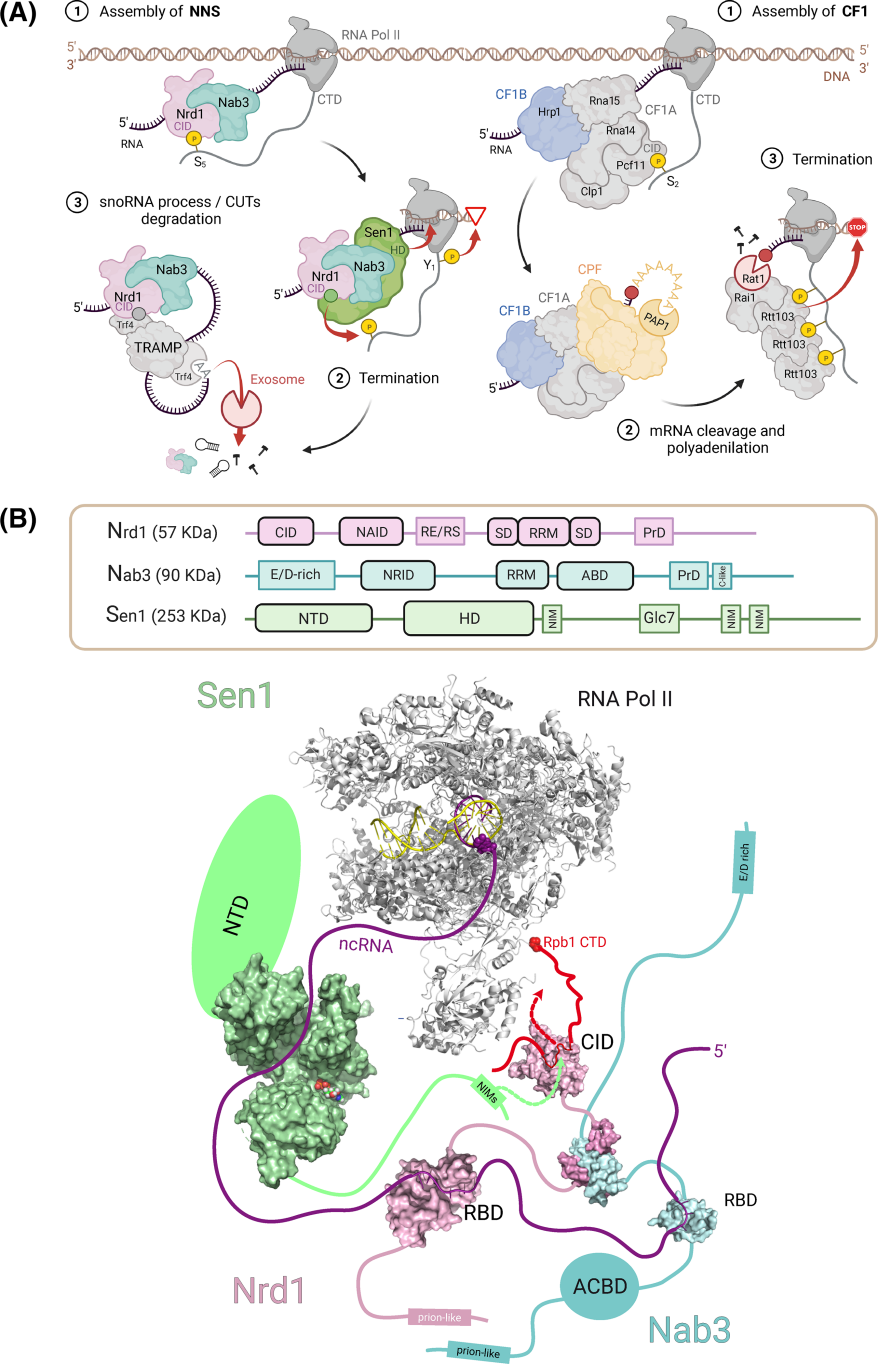
Mechanisms of RNA Pol II transcription termination. (**A**) Representation of the NNS and poly(A)-dependent termination pathways. Domain architectures of Nrd1, Nab3 and Sen1 are depicted at the bottom. (**B**) Graphical summary of the current structural knowledge of the NNS complex. The RNA polymerase II (PDB: 1Y1W) is shown in ribbon and Nrd1, Nab3 and Sen1 structures in surface (PDB codes in Supplementary Table S2) and color coded as in panel (**A**). Regions/domains without structural data available are represented as thick lines, rectangles or ellipses.

Transcription in animal/fungal cells is performed by three RNA polymerases, with RNA Pol II dealing with mRNAs and some non-coding RNAs (ncRNAs) [1,2]. RNA polymerases employ different termination pathways [3–5]. In the case of RNA Pol II transcription termination of mRNAs occur via the poly(A)-dependent pathway, (Figure 1 and reviewed in [2,6–9]), whereas CUTs [10–12] and snoRNAs [13–15] are terminated through the NNS complex in budding yeast. Additionally, the NNS promotes premature transcription termination of a handful of genes [16–18] and participates in the nuclear surveillance of aberrant mRNAs [19]. This complex, named after the proteins Nrd1 [13,20], Nab3 [21] and Sen1 [22], is early recruited to the transcription apparatus through the interaction between Nrd1 and phosphorylated Ser5 of the C-terminal repeats of RNA Pol II (hereafter CTD) [23,24]. The CTD is made up of 26 repeats of an heptapeptide (52 in humans) with the Y_1_S_2_P_3_T_4_S_5_P_6_S_7_ consensus sequence [25–29]. Then specific termination signals are recognized by the Nrd1/Nab3 heterodimer followed by the incorporation of the RNA helicase Sen1, which disconnects Nrd1 from the CTD and translocates along the nascent RNA to promote termination [30]. Subsequently, the TRAMP complex is recruited to the released transcripts to stimulate the action of the nuclear exosome [31], a multi-subunit complex that processes/degrades a variety of RNAs [32]. The exosome trims pre-snoRNAs to their mature version and fully degrades CUTs, diminishing the adverse effects of pervasive transcription [33]. However, in some cases the transcription of CUTs can regulate the expression of neighboring genes by a mechanism of transcriptional interference [34,35].

In this review, we recapitulate the current structural and biophysical knowledge of key interactions mediated by Nrd1, Nab3 and Sen1, and discuss about foresights in the structural biology field of the NNS pathway.

## Similarities between NNS and CFI complexes

Cleavage Factor I (CFI) is equivalent to the NNS complex in the poly(A)-dependent pathway (Figure 1A). These different complexes use a similar strategy based on the simultaneous recognition of specific RNA motifs in the transcript and specific phosphorylated patterns in the CTD. This dual recognition activates transcription pausing, disassembly of the RNA Pol II machinery and final processing of the RNA 3-end (reviewed in [2]). NNS and CFI complexes use RNA-binding proteins (RBPs) with sequence selectivity: Nrd1/Nab3 in NNS, and Hrp1/Rna15 in CFI. Structures of protein–RNA complexes have revealed the molecular basis of RNA selectivity [36–41]. Heterodimerization (Nrd1/Nab3), or interactions with a third partner (Hrp1/Rna15 with Rna14) (Figure 1) boost their selectivity enabling, a wide range of terminators to be recognized.

Nrd1 and Nab3 have a multidomain architecture with RRM-like, heterodimerization and prion-like domains (PrLD) [42–45] (Figure 1A), the latter important for the formation of nuclear granules [46]. In addition, Nrd1 has an N-terminal CTD-interacting domain (CID) for alternative recognition of CTD or Nrd1 Interaction Motifs (NIM) [24], while Nab3 has a predicted anticodon binding domain (ABD), probably involved in RNA binding. Sen1 is a larger protein with a central helicase domain (HD) [47], an N-terminal domain (NTD) that interacts with RNA Pol II [48,49] and Pol III [50], and a C-terminal intrinsically disordered region (IDR) that contains a functionally validated NIM [49] and two additional putative ones [51]. Finally, Nrd1 and Nab3 have other low-complexity regions whose functions are yet largely unknown.

## Nrd1, Pcf11 and Rtt103 CIDs as peptide recognition hubs

**Figure 2.**
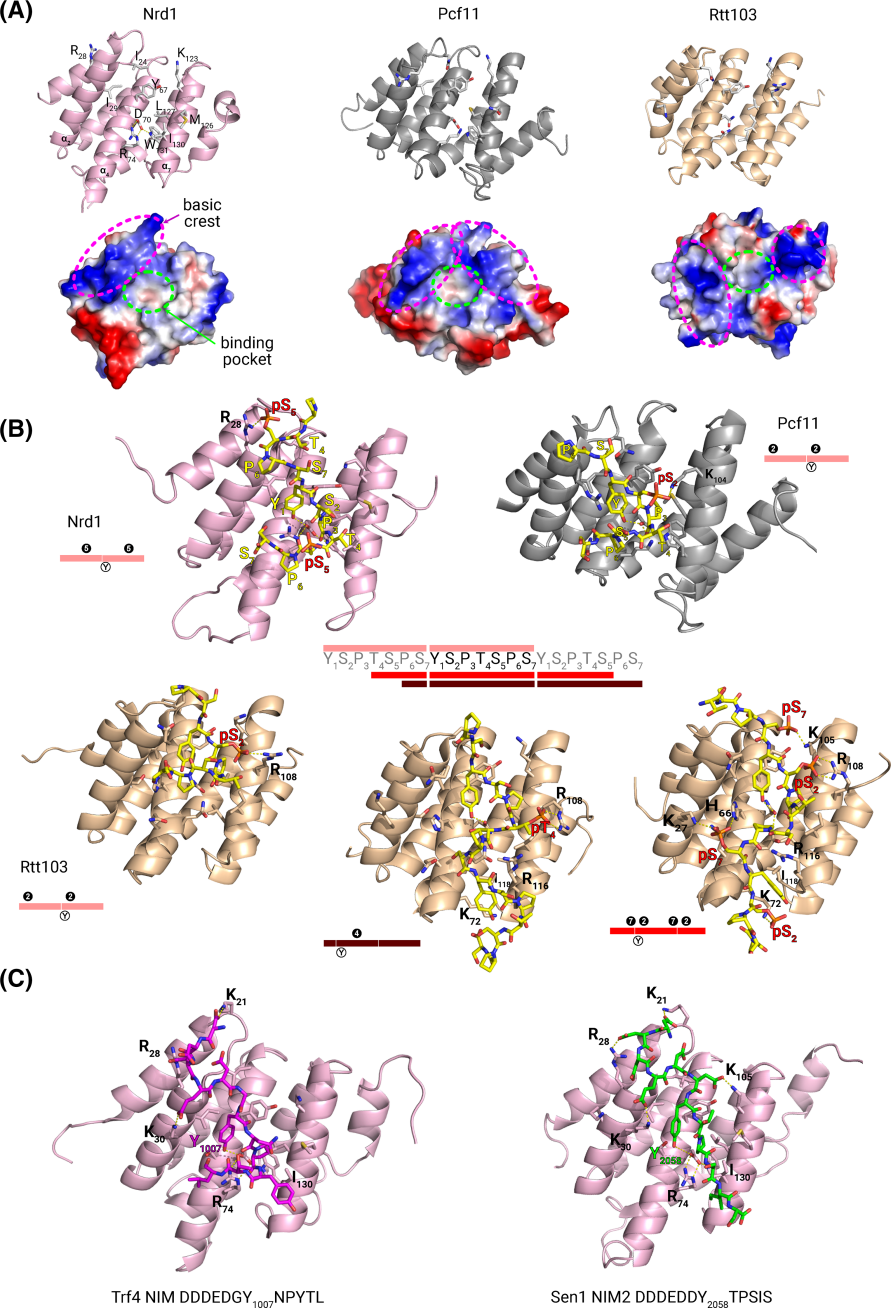
Structural summary of *Saccharomyces cerevisiae* CIDs. (**A**) Ribbon (above) and surface (below) representation of the structures of Nrd1 (light pink), Pcf11 (grey) and Rtt103 (light orange) CID. Charge distribution is mapped and key regions are circled in magenta (basic crest) or green (binding pocket). Residues involved in protein–protein recognition are labeled on Nrd1 CID. (**B**) Structural details of different RNA Pol II CTD phosphopeptides recognized by Nrd1, Pcf11 and Rtt103 CIDs (color codes as in panel **A**). Peptide frameworks are marked in different shades of red besides a sequence containing three heptapeptide repeats. The phosphorylation patterns are encircled next to the structures. (**C**) Structures of two representative Nrd1 CID–NIM complexes. PDB codes in Supplementary Table S2.

The Nrd1 CID is a ∼150-residue-long α-helical domain with the second, fourth and seventh helices defining the binding pocket depression and a positively charged flanking crest (Figure 2A) [23]. The earlier structure of Pcf11 CID [52,53], a CFI complex component [54,55], and the Rtt103 CID structure [56], a Rat1 exonuclease associated transcription termination factor [57], share the same armadillo-repeats like fold, but with different charge distributions around the binding pocket (Figure 2A).

Nrd1, Pcf11 and Rtt103 CIDs interact with CTD phosphorylated Ser/Thr forms much better than with unphosphorylated [56,58,59] or CTD-pTyr1 ones [58] (Supplementary Table S1). Pcf11 and Rtt103 recognize preferentially CTD-pSer2 peptides [56,58], with Rtt103 binding tighter (*K*_D_s = 2–15 µM) than Pcf11 (70–160 µM). Rtt103 also binds CTD-pSer2/7 (1.6 µM) [60] and CTD-pThr4 (6 µM) [61] with comparable affinity. Conversely, Nrd1 CID prefers CTD-pSer5 peptides (*K*_D_s = 40–216 µM) [23,24,51,58,59] over other marks [56], and binds the CTD-pSer2/5 dual mark slightly better (16 µM) [23]. Overall, these biophysical data are consistent with ChIP data [58] and suggest that Nrd1 is recruited to transcription earlier than Pcf11/Rtt103.

The binding pockets of all CID–CTD complexes (Figure 2B) recognize CTD-Tyr1 and Pro3 through conserved hydrophobic/polar contacts. The CID geometries are preconfigured in the free states of Nrd1 and Pcf11 by ion pairs/hydrogen bonds between Arg74/Asp70 in Nrd1, and Lys72/Asp68 in Pcf11 (Figure 2). CTD-Tyr1 recognition is achieved by a key hydrogen bond between its OH and Asp/Asn residues in helix 4, that would be not possible in CTD-pTyr1. CTD-Pro3 interacts with a conserved Tyr in helix 4 and two hydrophobic side-chains in helix 7 (Figure 2A,B) and, in many complexes, the CTD Pro3 forms a β-turn. In general, peptide bonds of CTD-Pro3/6 adopt the *trans* configuration (SCAF4 and SCAF8 complexes included [62,63])*,* with the exception of the Nrd1 CID/CTD-pSer5 complex where the pSer5-Pro6 bond of the first repeat is in *cis*, causing a strong kink [59]. A similar case was found in the Ssu72/CTD-pSer5 complex [64,65].

Negatively charged pSer/pThr are solvent-exposed and recognized by positively charged Lys and Arg residues (Figure 2B). When using CTD peptides with two complete diphosphorylated repeats (either pSer2 or pSer5), the structures of Nrd1, Pcf11 and Rtt103 complexes just show the recognition of one of the phosphates: Arg28, in Nrd1 helix 2, interacts with the first pSer5 of the CTD [59] and Lys104, in Pcf11 helix 7, with the second pSer2 [52], an equivalent contact to that between Rtt103 Arg108 and pSer2 [56] (Figure 2B). These three complexes present the characteristic β-turn after CTD Pro3 with the backbone folding towards helix 2. However, using slightly longer CTD peptides and, more importantly, with different frame (Figure 2B), allows the recognition of Tyr1 in the third repeat, inducing the peptides to follow a different route along the helix 4/7 interface. A similar peptide conformation occurs in the Rtt103 and CTD pSer2/pSer7[60] or CTD pThr4 [61] complexes (Figure 2B).

Nrd1 CID also interacts with NIMs (∼12-residue peptides), the first one discovered in Trf4, the poly(A) polymerase of the TRAMP complex [24]. These data add structural insights to previous biochemical studies that established the relationship between the NNS complex and nuclear exosome [66]. Further NIMs were later found in Mpp6 [67], an exosome cofactor, and in Sen1 [49,51]. The NIMs show a distinct pattern of negatively charged residues preceding the tripeptide Yx(P/L) and bind to Nrd1 CID stronger than CTD-pSer5 (*K*_D _= 40–216 µM): *K*_D_ of 0.9–5.7 µM for Trf4 NIM [24,51,67]; 1.6–1.9 µM for Sen1 NIM2 [49,51] and 13.6 µM for Mpp6 [67]. The structures of Nrd1 in complex with Trf4 and Sen1 NIMs illustrate the fundamentals of the interaction (Figure 2C). The Tyr-x-Pro motif interacts as in the CID–CTD complexes (Figure 2B) and the N-terminal acidic residues form ion-pairs with the Lys/Arg at the Nrd1 CID basic crest (Figure 2A,C). C-terminal residues are recognized by the lower part of helices 4 and 7, like in Rtt103 complexes with CTD-pThr4 and CTD-pSer2/7 (Figure 2B).

## Nrd1/Nab3 RNA recognition

**Figure 3.**
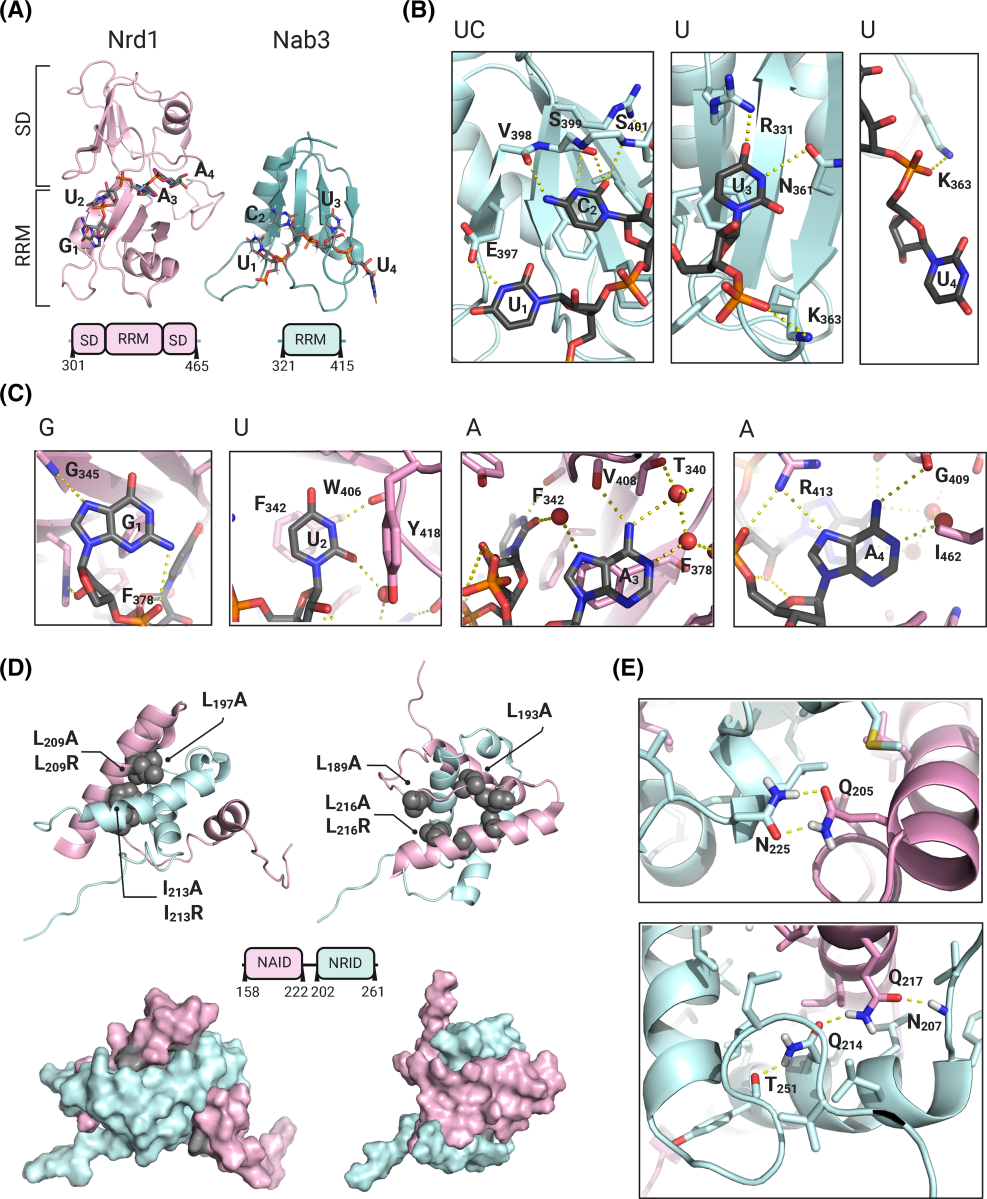
RNA-binding specificity of Nrd1 and Nab3 RBDs and structural basis of Nrd1/Nab3 heterodimerization. (**A**) Complex structures between Nrd1 (light cyan ribbon) and Nab3 (light pink ribbon) RBDs and GUAA and UCUU motifs (sticks). (**B**) Atomic details of the Nab3 RRM and UCUU interaction. Protein residues involved in RNA contacts are shown as sticks and key hydrogen bonds involved in base specificity as dashed yellow lines. (**C**) Detailed view of the Nrd1–GUAA interactions involved in base specificity. Key structural water molecules are shown as spheres. (**D**) Ribbon representation of an Nrd1–Nab3 chimera representative of the heterodimer. Nrd1 and Nab3 are color coded as in **A** and **B**. Buried Leu and Ile residues mutated to Ala in functional studies are represented as atom spheres. (**E**) Detailed view of the two hydrogen bond networks that lock the conformation on the structure (Nrd1 Q205:Nab3 N225 and Nrd1 Q217:Nab3 Q214). PDB codes in Supplementary Table S2.

Nrd1 [20] and Nab3 [68] contain RNA Recognition Motifs (RRMs) that interact with ssRNA (Figure 3A) [69–72]. The Nrd1 binding site was early mapped to the UGUAAA sequence in the U6 transcript [73]. Later, the discovery of Nrd1/Nab3 heterodimerization suggested that the two factors combine their selectivity to recognize longer RNAs [68]. A first milestone linked these proteins to a novel poly(A)-independent mechanism for the formation of the 3′ end of snoRNAs [13]. The biophysical characterization of NNS terminators in various snoRNA genes provided binding affinity data for the heterodimer and define consensus sequences for Nrd1/Nab3 binding sites [74,75], which were soon identified also in CUTs [10,12,76]. Afterwards, an *in vivo* SELEX study [77] set the optimal Nrd1 and Nab3 binding motifs to A/UGUAAA and UCUUG. Finally, PAR-CLIP [78,79], and CRAC [80] methods have been used to map the Nab3, Nrd1 and Sen1 binding sites *in vivo*. These transcriptome-wide experiments showed an enrichment of previously identified motifs (e.g. UCUU, CUUG for Nab3 and GUAA, GUAG for Nrd1) [78–80], other related (GNUUCUGU for Nab3 and UGUAG for Nrd1) [78] and even new purine-rich ones (UGGA, GAAA for Nrd1) [80].

Nrd1 and Nab3 protein–RNA complexes have been studied by structural and biophysical methods. The structure of the Nab3 RRM/UCUU complex has been solved by NMR and X-ray (Figure 3A) [39,40] and binding affinities in the 48–110 µM range have been measured for snR47 and snR13 terminators (containing two UCUU repeats). However, the inclusion of a 40-residue extension at the N-terminus of Nab3 RRM seems to discriminate the high affinity pentameric site AUCUUGA (36.7 ± 2.4 µM) over the tetrameric one AUCUUCA (165.6 ± 6.1 µM) [77], probably through structural rearrangements in this extra element [76]. In Nab3 complexes, the UCUU motif adopts a singled-stranded conformation stabilized by specific contacts with three of the bases (Figure 3A,B). The C2 base moiety stacks with the Phe333 aromatic ring and makes base-specific contacts with the main chain carbonyl groups of Val398 and Ser400, and with the hydroxyl group of Ser399 (Figure 3B) [39]. The imino groups of U1 and U3 are specified by hydrogen bonds to Glu397 carboxyl and Asn361 carbonyl side-chains. Further residues are involved in hydrophobic contacts with sugars (i.e. Ile395, Phe366) and ion pairs with phosphates (i.e. Lys363) (Figure 3B). The X-ray structure does not shed light on U4 recognition, but the NMR one points to the presence of specific contacts with the Asn364 side-chain.

NMR and X-ray structures of the Nrd1 RNA-binding domain (RBD) reveal an unusual arrangement [41] (Figure 3A). The canonical RRM fold is flanked by N/C-terminal regions that assemble together to form a subdomain dubbed as ‘split domain' (SD). The SD scaffold is fused to the RRM and built by a mixed β-sheet and an α-helix perpendicular to it. Interestingly, a former Nrd1 structure showed partially folded regions flanking the RRM [81], which are consistent with NMR spectral changes observed in samples produced above 12°C [41]. The functional implications of this temperature-sensitive folding remain to be determined. Nrd1 RBD binds CCGUAACC and CCGUAGCC RNAs with 2.1 ± 0.1 µM and 8 ± 1 µM *K*_D_ values [41], and the complexes with GUAA/CGUAAA/UUUAGUAAUCC confirm the involvement of both RRM and SD in the core GUAA recognition [41] (Figure 3A,C). The RRM domain interacts with the first three nucleotides with U2 and A3 forming archetypal planar stacking interactions with Phe342 and Phe378, whereas G1 stacks to the edge of the Phe342 ring (Figure 3C). SD domain residues Ile369 and Tyr418 make additional stacking interactions with U2 and A3, while His303, Ile462 and Val464 make further contacts with A4. The RNA specificity is explained by direct hydrogen bonds of G1 with Gly345 and Arg403, U2 with Trp406 and Tyr418, and adenines A3 and A4 with Val408, Gly409 and Arg413 (Figure 3C). The protein/RNA interface encloses a water pocket between the RRM and SD with several structural water molecules mediating A3/A4 recognition. Other bases flanking the 4-mer core (in complexes with CGUAAA and UUUAGUAAUCC) do not show direct contact with the protein but are loosely stabilized by intra-RNA contacts.

## Nrd1/Nab3 heterodimerization

Nrd1/Nab3 heterodimerization promotes high affinity binding to NNS terminators [68] and multiple copies of Nrd1 and Nab3 would assemble on non-poly(A) terminators through different types of cooperative interactions among these proteins [75]. A recent study reveals the atomic details of Nrd1/Nab3 heterodimerization [82]. In the free states, the Nrd1 Interaction Domain (NRID) of Nab3 forms a helical structure without a defined tertiary fold, whereas the Nab3 Interaction Domain (NAID) of Nrd1 seems to form a helical oligomer (as derived from CD and NMR data). The NMR structure of an Nrd1–Nab3 chimera (modeling the heterodimer) shows an unusual α-helical arrangement where Nab3 forms the core of the structure while Nrd1 fastens around it locking an unique conformation of the NRID (Figure 3D). The large burial of hydrophobic residues (Ile, Phe, Val and Leu) at the Nrd1/Nab3 interface likely explains the nanomolar affinity of the interaction [56,82]. The structure is defined by two conserved (and buried) hydrogen bond networks involving Nab3 Asn225-Nrd1 Gln205 and Nab3 Gln214-Nrd1 Gln217 (Figure 3D). Phenotypic studies showed that Nrd1 NAID tolerates Leu/Ile to Ala substitutions but not replacement by structural-destabilizing Arg residues (Figure 3D). These structure-guided functional studies confirm the relevance of Nrd1/Nab3 heterodimerization in the NNS pathway and provide specific tools to study its mechanistic contribution.

## Sen1 is a key transcription termination factor

Sen1 is the largest and most important protein factor in NNS termination. This RNA helicase is homologous to human senataxin (SETX), a critical gene linked to neurological disorders and involved in transcription termination and R-loops regulation [83]. Sen1 interacts with ssRNA/ssDNA using its ATP-dependent RNA HD, with affinities in the nanomolar to submicromolar range [34,47]. Several reports showed that Sen1 lacks nucleic acid sequence selectivity [30,78,79], although one study found that Sen1 HD pulls-down bacterial RNAs rich in the (CAN)_4_ motif during its purification [84]. Sen1 has a 5′–3′ RNA unwinding activity [84–86] and can promote transcription termination [30]. In contrast with Upf1, Sen1 has low processivity on RNA translocation, a feature that, together with its low abundance, is important to control its termination activity [86]. The X-ray structure of the ADP-bound Sen1 HD (Figure 1) confirms an equivalent domain composition to Upf1-like helicases [47]. The two RecA domains are in an open conformation sandwiching the ADP nucleotide and define part of the RNA-binding channel. The RecA1 domain has two accessory subdomains: 1B includes the ‘stalk' and the ‘β-barrel' and 1C is defined as the ‘prong'. An N-terminal extension (the ‘brace') interacts with subdomain 1B restricting its spatial sampling capabilities in comparison with other Upf1-like helicases. A similar conformation of the β-barrel is found in Upf1 forced by the interaction with an accessory domain [87]. In the Sen1 HD structure only the lower part of the ‘prong' is visible, presumably because high mobility of the upper part [47]. Biochemical studies showed that this element is critical for transcription termination [47,86].

**Figure 4.**
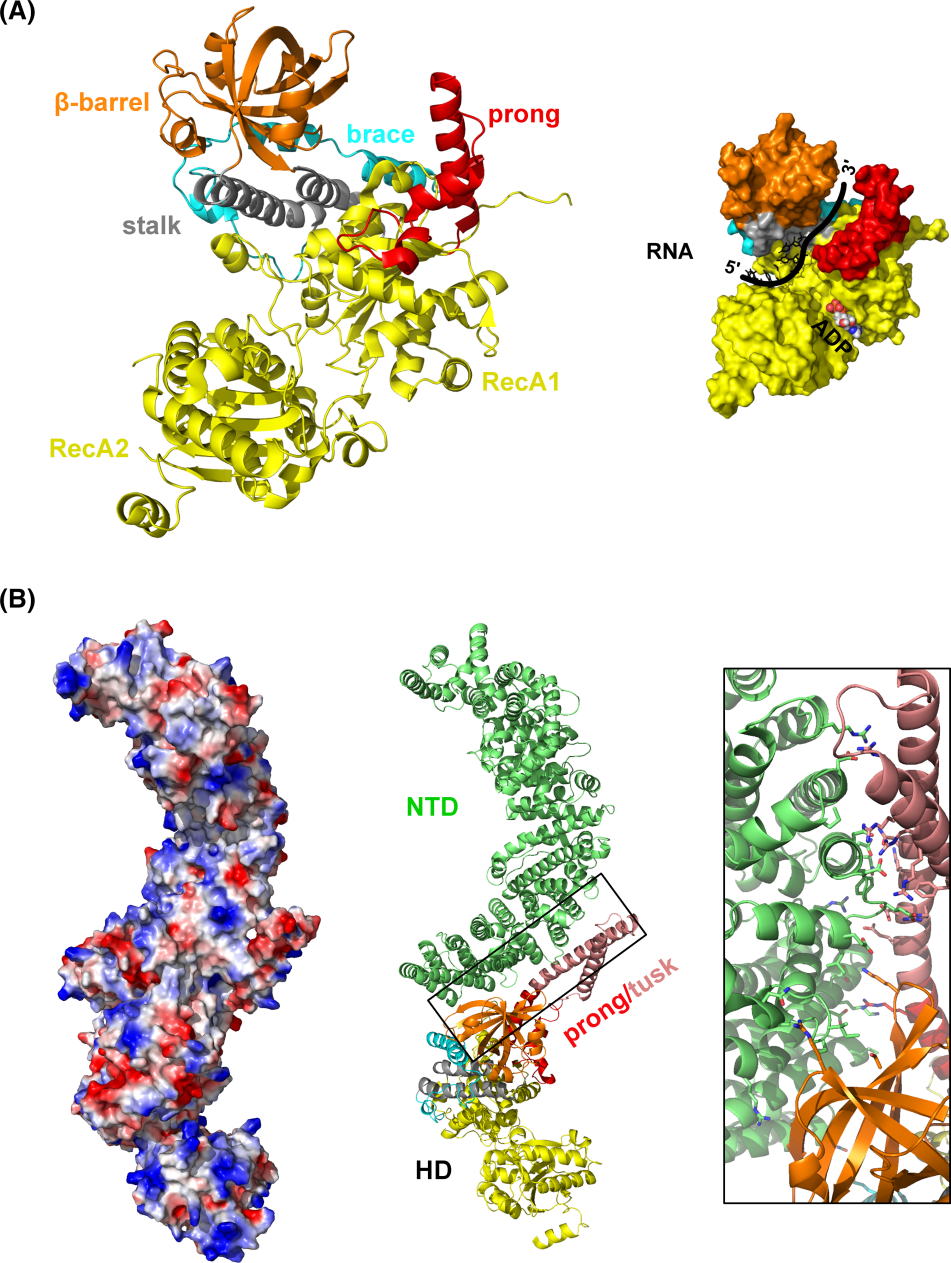
Sen1 structural information. (**A**) Ribbon representation of the X-ray structure of Sen1 helicase domain (PDB: 5MZN) with the domains/subdomains colored as in [47]. On the right a surface model of the proposed RNA-binding mode is depicted. The first five nucleotides have been taken from the structure of Upf1-RNA (PDB:2XZL [87]). The predicted orientation of the RNA is indicated as a thick black line. (**B**) AlphaFold structure of Sen1 (disordered regions have been removed for clarity). Domains and subdomains of Sen1 HD have been colored as in the panel **A**, with the exception of the upper part of the prong that is colored in salmon and is dubbed here as ‘tusk'. The Sen1 NTD is represented in green and a detail of the HD/NTD interface is shown on the right. A surface charge distribution is shown on the left with the molecule in the same orientation.

In addition to the HD, Sen1 has an N-terminal domain (NTD) that has been involved in interactions with Pol II [48] and Pol III [50]. Although the NTD structure is yet experimentally unknown, an AlphaFold [88] model predicts that it is composed by HEAT-like helical repeats forming a super helical solenoid-like structure (Figure 4B). Interestingly, the NTD interacts with the HD through a large interface that seems to alter the structure of the ‘prong'. In the Alphafold structure the upper part of the ‘prong' forms a two-helical hairpin that we name ‘tusk', that protrudes out and is negatively charged. Following the importance of this element for termination [47], it is tempting to speculate that the protruding may interfere with the RNA/DNA duplex at the transcription bubble, resembling the mechanism of termination proposed for 3′–5′ helicases (discussed in [47]). Alternatively, because Sen1 NTD has been shown to negatively regulate termination [86], it is possible that the Alphafold structure actually represents an inhibitory conformation of the ‘prong'. Whatever the case it is clear that further experiments on Sen1 are needed to unveil its mechanism. In the meantime, the Alphafold structure can help to propose new hypotheses and design biochemical experiments to test them.

Biophysical and structural data complement genetic and biochemical studies to propose an integrated NNS mechanism (Figure 1A). Nrd1/Nab3 would be recruited shortly after transcription initiation by CTD-pSer5 recognition and/or binding to NNS terminators. Sen1, responsible for transcription termination, is recruited via the NIM–Nrd1 CID. RNA Pol II pausing is required for ncRNA termination [30], perhaps caused by Nrd1/Nab3 RNA binding and/or, as recently proposed, by CTD Tyr1 phosphorylation [89]. After termination, TRAMP would be recruited by the Nrd1–Trf4 interaction [31]; Trf4 would synthetize short poly(A) tails at the 3’-end that target the transcripts for processing (snoRNAs) [90] or degradation (CUTs) by the exosome. Exosome activity is required for the physical release of Nrd1 and Nab3 from the RNA [91].

## Intrinsically disordered regions

Nrd1, Nab3 and Sen1 have large IDRs containing short protein binding motifs, large low complexity/prion-like domains and homodimerization motifs (Figure 1). In addition to the NIMs [49,51], the Sen1 IDR includes a Glc7 (the phosphatase of the Cleavage and Polyadenylation Factor (CPF) complex) binding site [92]. Nab3 has a 200-long N-terminal acidic IDR of unknown function that seems dispensable. In contrast, both Nab3 and Nrd1 have P/Q rich C-terminal PrLD of ∼240 and 110 residues [45] whose deletions cause slow growth phenotypes [43,74]. The Nab3 PrLD forms fibers and hydrogels [93] and is required for the accumulation of nuclear-periphery granules upon glucose depletion [46], likely formed by liquid–liquid phase separation (LLPS). The last 18 residues of this domain are highly homologous to the oligomerization domain of hnRNP C [42,94].

## Outlook

Several structural aspects of the NNS complex need to be experimentally addressed in the future; perhaps the most important is related to the structure and interactions of Sen1 N-terminal domain. The versatility of Nrd1 CID in the recognition of non-CTD peptides suggests that Pcf11 and Rtt103 CID might also be able to interact with non-CTD peptides yet to be discovered. Continuing with Nrd1, its particular RBD architecture is conserved in SCAF4 and SCAF8 AlphaFold models, suggesting, that apart from RNA binding, these domains could play a role in other interactions yet to be identified; perhaps with other regions of RNA Pol II?

Coming to Nab3, the structure and function of its ABD has not yet been studied. It is tempting to investigate if this domain is involved in the processing of RNA Pol III products.

Finally, the importance of Nab3 nuclear granules [95] deserves further investigations (also for Nrd1 PrLD) and anticipates new roles of NNS in the regulation of the stress response.

## Perspectives

- The Nrd1, Nab3, Sen1 (NNS) pathway performs, in budding yeast, transcription termination of ncRNAs like snoRNA and CUTs. Understanding the molecular interactions between its components and with other parts of the transcription machinery is critical to propose mechanistic models.
- Nrd1, Nab3 and Sen1 are RNA-binding proteins with modular architectures and capability to interact with peptide motifs (i.e. Nrd1-CTD/NIM interactions), heterodimerize (Nrd1/Nab3) or oligomerize (Nrd1/Nab3 PrLDs). Many of these interactions have been structurally and biophysically characterized providing important insight into the NNS mechanism.
- There are still missing pieces to unravel the NNS structural puzzle, including the structure and interactions of Nab3 ABD and Sen1 NTD domains and the biomolecular condensation properties of Nrd1/Nab3 low-complexity regions involved in the formation of nuclear granules upon stressors.

## Abbreviations

ABD: anticodon binding domain
CFI: Cleavage Factor I
ChIP: chromatin immunoprecipitation
CID: CTD-interacting domain
CUTs: cryptic unstable transcripts
HD: helicase domain
IDR: intrinsically disordered region
NAID: Nab3 interaction domain
NIM: Nrd1 interaction motifs
NMR: nuclear magnetic resonance
NNS: Nrd1/Nab3/Sen1
NRID: Nrd1 interaction domain
NTD: N-terminal domain
PrLD: prion-like domains
RBD: RNA-binding domain
SD: split domain
TRAMP: Trf4/5 Air1/2 Mtr4 polyadenylation complex
