# Supplementary material for: The Nrd1–Nab3–Sen1 transcription termination complex from a structural perspective
Source: Biochem Soc Trans. 2023 May 24;51(3):1257–69. doi: 10.1042/BST20221418 (PMC10317158; doi:10.1042/BST20221418)
Supplement: Supplementary Material 1 [file BST-51-1257-s1.pdf]

Supplementary data for
The Nrd1-Nab3-Sen1 transcription termination complex from a structural perspective

Belén Chaves-Arquero[1]* and José Manuel Pérez-Cañadillas[2]*

Peptide/RNA Sequence	Length	Technique	BUFFER	Temperature(ºC)	K_D(µM)	K_D error	REF
YSPT**S**PSYSPT**S**PS	14	FA (competition)	50 mM HEPES(pH 7.5), 150 mM NaCl, 1 mM EDTA and 1 mM DTE	10	40	3	23
Y**S**PTSPSY**S**PTSPS	14	FA (competition)	50 mM HEPES(pH 7.5), 150 mM NaCl, 1 mM EDTA and 1 mM DTE	10	390	30	23
Y**S**PT**S**PSY**S**PT**S**PS	14	FA (competition)	50 mM HEPES(pH 7.5), 150 mM NaCl, 1 mM EDTA and 1 mM DTE	10	16	1	23
SPT**S**PSYSPT**S**PS	13	FA (competition)	20 mM Tris (pH 7.0), 200mMNaCl, 5mMDTT	RT	216,4	55,9	51
YSPT**S**PSYSPT**S**PS	14	FA	50mM NaPi (pH=8.0); 100 mM NaCl	10	131,24	13,17	24
YSPT**S**PSYSPT**S**PS	14	FA	50mM NaPi (pH=8.0); 100 mM NaCl	10	113	5	59
YSPT**S**P**S**YSPT**S**P**S**	14	FA	50mM NaPi (pH=8.0); 100 mM NaCl	10	140	20	59
YSPTSPSYSPTSPS	14	FA	50mM NaPi (pH=8.0); 100 mM NaCl	10	700	100	59
PSYSPT**S**PSYSPT**S**PS	16	FA (competition)	150 mM NaCl	25?	85	25	58
Y**S**PTSPSY**S**PTSPS	14	FA (competition)	25 mM HEPES (pH 8.0), 100mM NaCl, 1 mM EDTA, 1 mM DTE	25	130	35	56
Y**S**PTSPSY**S**PTSPS	14	NMR	10 mM Tris-HCl, (pH 7.4), 120 mM NaCl; 2 mM DTT	25	160	50	56
YSPTSPSYSPTSPS	14	FA (competition)	25 mM HEPES (pH 8.0), 100mM NaCl, 1 mM EDTA, 1 mM DTE	25	>1000		56
YSPTSPSYSPTSPS	14	NMR	10 mM Tris-HCl, (pH 7.4), 120 mM NaCl; 2 mM DTT	25	1500	300	56
YSPT**S**PSYSPT**S**PS	14	FA (competition)	25 mM HEPES (pH 8.0), 100mM NaCl, 1 mM EDTA, 1 mM DTE	25	>1000		56
YSPT**S**PSYSPT**S**PS	14	NMR	10 mM Tris-HCl, (pH 7.4), 120 mM NaCl; 2 mM DTT	25	1200	500	56
Y**S**PT**S**PSY**S**PT**S**PS	14	FA (competition)	25 mM HEPES (pH 8.0), 100 mM NaCl, 1 mM EDTA, 1 mM DTE	25	370	"+160/-110"	56
Y**S**PT**S**PSY**S**PT**S**PS	14	NMR	10 mM Tris-HCl, (pH 7.4), 120 mM NaCl; 2 mM DTT	25	630	170	56
SY**S**PTSPSY**S**PTSPS	15	FA (competition)	150 mM NaCl	25?	70	18	58
SY**S**PTSPSY**S**PTSPS	15	FA (competition)	10 mM NaCl	25?	54	12	58
YSPTSPSYSPTSPS	14	FA (competition)	25 mM HEPES (pH 8.0), 100mM NaCl, 1 mM EDTA, 1 mM DTE	25	>1000		56
YSPTSPSYSPTSPS	14	NMR	10 mM Tris-HCl, (pH 7.4), 120 mM NaCl	25	1200	300	56
Y**S**PTSPSY**S**PTSPS	14	FA (competition)	25 mM HEPES (pH 8.0), 100mM NaCl, 1 mM EDTA, 1 mM DTE	25	2,1	0,1	56
Y**S**PTSPSY**S**PTSPS	14	NMR	10 mM Tris-HCl, (pH 7.4), 120 mM NaCl	25	15	10	56
Y**S**PTSPSYSPTSPS	14	FA (competition)	25 mM HEPES (pH 8.0), 100mM NaCl, 1 mM EDTA, 1 mM DTE	25	nd	nd	56
Y**S**PTSPSYSPTSPS	14	NMR	10 mM Tris-HCl, (pH 7.4), 120 mM NaCl	25	420	79	56
YSPTSPSY**S**PTSPS	14	FA (competition)	25 mM HEPES (pH 8.0), 100mM NaCl, 1 mM EDTA, 1 mM DTE	25	nd	nd	56
YSPTSPSY**S**PTSPS	14	NMR	10 mM Tris-HCl, (pH 7.4), 120 mM NaCl	25	76	16	56

YSPT**S**PSYSPT**S**PS	14	FA (competition)	25 mM HEPES (pH 8.0), 100mM NaCl, 1 mM EDTA, 1 mM DTE	25	122	3	56
YSPT**S**PSYSPT**S**PS	14	NMR	10 mM Tris-HCl, (pH 7.4), 120 mM NaCl	25	600	300	56
Y**S**PT**S**PSY**S**PT**S**PS	14	FA (competition)	25 mM HEPES (pH 8.0), 100mM NaCl, 1 mM EDTA, 1 mM DTE	25	53	10	56
Y**S**PT**S**PSY**S**PT**S**PS	14	NMR	10 mM Tris-HCl, (pH 7.4), 120 mM NaCl	25	41	19	56
TSP**S**Y**S**PTSP**S**Y**S**PTS	16	FA	35 mM KPI (PH 6.8), 100 mM KCl	10	1,6	0,1	60
PSYSP**T**SPSYSP**T**SPS	16	FA	35 mM KPI (PH 6.8), 100 mM KCl	10	6	0,2	61
PSYSPTSPSYSPTSPS	16	FA	35 mM KPI (PH 6.8), 100 mM KCl	10	43	2	61
PSY**S**PTSPSYSPTSPS	16	FA	35 mM KPI (PH 6.8), 100 mM KCl	10	6	0,2	61
SY**S**PTSPSY**S**PTSPS	15	FA (competition)	150 mM NaCl	25?	12	2	58
gggAAAACGAAAUAAAUCUC UUUGUAAAACGGUUCAUCC UUAU	43	EMSA	100 mM NaCl, 20 mM Tris·HCl, pH 8.0, 10% glycerol, 0.1 mM DTT and 0.1 mM EDTA		0,01		73
gggAAAACGAAAUAAAUCUC UUUGUAAAACGGUUCAUCC UUAU	43	EMSA	100 mM NaCl, 20 mM Tris·HCl, pH 8.0, 10% glycerol, 0.1 mM DTT and 0.1 mM EDTA		0,1		73
gggAAAACGAAAUAAAUCGC UUUGUAAAACGGUUCAUCC UUAU	43	EMSA	100 mM NaCl, 20 mM Tris·HCl, pH 8.0, 10% glycerol, 0.1 mM DTT and 0.1 mM EDTA		0.04-0.05		73
AUCUUGA	7	FA	water	25	36.7	2.4	77
AUCUUCA	7	FA	water	25	165.6	6.1	77
CCGUAACC	8	ITC	20 mM KPi pH 7.0, 150 mM NaCl and 2 mM B-mercaptoethanol	25	2.1	0.1	41
CCGUAGCC	8	ITC	20 mM KPi pH 7.0, 150 mM NaCl and 2 mM B-mercaptoethanol	25	8	1	41
CCGUAACC	8	FA	20 mM Tris-HCl, 150 mM NaCl and 1 mM DTT pH 8.0	26	1.4	0.2	41
CCGUAGCC	8	FA	20 mM Tris-HCl, 150 mM NaCl and 1 mM DTT pH 8.0	26	5	1	41
GUAA	4	FA	150 mM NaCl and 10 mM beta-mercaptoethanol pH 8.0	20	10,1	0,8	81
UGGA	4	FA	150 mM NaCl and 10 mM beta-mercaptoethanol pH 8.0	20	94,1	3,9	81
UCCU	4	FA	150 mM NaCl and 10 mM beta-mercaptoethanol pH 8.0	20	>500		81
UCUUG	5	FA	150 mM NaCl and 10 mM beta-mercaptoethanol pH 8.0	20	41,1	2,7	81
GGGGGGG	7	FA	150 mM NaCl and 10 mM beta-mercaptoethanol pH 8.0	20	5,7	1,2	81
UUUUUUU	7	FA	150 mM NaCl and 10 mM beta-mercaptoethanol pH 8.0	20	69,8	3,2	81
AAAAAAA	7	FA	150 mM NaCl and 10 mM beta-mercaptoethanol pH 8.0	20	32,1	0,9	81
CCCCCCC	7	FA	150 mM NaCl and 10 mM beta-mercaptoethanol pH 8.0	20	>500		81
AUAUAUA	7	FA	150 mM NaCl and 10 mM beta-mercaptoethanol pH 8.0	20	11,3	0,5	81
AUUAUUA	7	FA	150 mM NaCl and 10 mM beta-mercaptoethanol pH 8.0	20	14,8	0,4	81
GUUGUUG	7	FA	150 mM NaCl and 10 mM beta-mercaptoethanol pH 8.0	20	13	0,7	81
UGGUGGU	7	FA	150 mM NaCl and 10 mM beta-mercaptoethanol pH 8.0	20	6,8	0,6	81
GCGGGCG	7	FA	150 mM NaCl and 10 mM beta-mercaptoethanol pH 8.0	20	13,2	0,5	81

GCGGGGC	7	FA	150 mM NaCl and 10 mM beta-mercaptoethanol pH 8.0	20	9,8	0,3	81
CAGCGUC	7	FA	150 mM NaCl and 10 mM beta-mercaptoethanol pH 8.0	20	37,7	1,1	81
CACACAC	7	FA	150 mM NaCl and 10 mM beta-mercaptoethanol pH 8.0	20	108,5	4	81
GUAAUGAAUUAAGUCUUG AUAUAUAA	26	FA	150 mM NaCl and 10 mM beta-mercaptoethanol pH 8.0	20	1,5	0,1	81
AGCGUGAAUUAAGUCUUG AUAUAUAA	26	FA	150 mM NaCl and 10 mM beta-mercaptoethanol pH 8.0	20	2,5	0,2	81
AGCGUGAAUUAAGAGCGUA UAUAUAA	26	FA	150 mM NaCl and 10 mM beta-mercaptoethanol pH 8.0	20	7,9	0,3	81
AGCGUGAAUUAAGUCUUGC ACACACA	26	FA	150 mM NaCl and 10 mM beta-mercaptoethanol pH 8.0	20	5,4	0,2	81
CCCCUGAAGCCCCCCCCCCC CC	26	FA	150 mM NaCl and 10 mM beta-mercaptoethanol pH 8.0	20	7,2	0,5	81
UUCUUAUUCUUA	12	FA	10 mM Hepes-NaOH pH 7.3, 150 mM NaCl and 1 mM dithioerythritol	25	110	20	56
UUCUUAUUUUUA	12	FA	10 mM Hepes-NaOH pH 7.3, 150 mM NaCl and 1 mM dithioerythritol	25	100	20	56
UUUUUAUUUUUA	12	FA	10 mM Hepes-NaOH pH 7.3, 150 mM NaCl and 1 mM dithioerythritol	25	350	140	56
UCCCUAUCCCUA	12	FA	10 mM Hepes-NaOH pH 7.3, 150 mM NaCl and 1 mM dithioerythritol	25	250	60	56
UUCUUAUUCUUA	12	FA	10 mM Hepes-NaOH pH 7.3, 150 mM NaCl and 1 mM dithioerythritol	25	70	20	56
UUCUUAUUUUUA	12	FA	10 mM Hepes-NaOH pH 7.3, 150 mM NaCl and 1 mM dithioerythritol	25	70	20	56
UUUUUAUUUUUA	12	FA	10 mM Hepes-NaOH pH 7.3, 150 mM NaCl and 1 mM dithioerythritol	25	470	130	56
UCCCUAUCCCUA	12	FA	10 mM Hepes-NaOH pH 7.3, 150 mM NaCl and 1 mM dithioerythritol	25	230	40	56
UUUUCUUUUUUCUUAUUC UUAUU	23	FA	50 mM NaPi pH 7.5, 150 mM NaCl and 10 mM beta-mercaptoehanol	25	48	2	40
CCCCGUAGAAAAUCUUAGU AAUCCUUCUUACAUUG	35	FA	50 mM NaPi pH 7.5, 150 mM NaCl and 10 mM beta-mercaptoehanol	25	46	1	40
GGUAGUAUAACAAUAUCCG UGUUGUUAUAGUACC	35	FA	50 mM NaPi pH 7.5, 150 mM NaCl and 10 mM beta-mercaptoehanol	25	1424	198	40
UCUU	4	FA	50 mM NaPi pH 7.5, 150 mM NaCl and 10 mM beta-mercaptoehanol	25	763	23	40
GUAA	4	FA	50 mM NaPi pH 7.5, 150 mM NaCl and 10 mM beta-mercaptoehanol	25	4726	591	40
CCCCGUAGAAAAUCUUAGU AAUCCUUCUUACAUUG	35	FA	50 mM NaPi pH 7.5, 150 mM NaCl and 10 mM beta-mercaptoehanol	25	11	1	40
GUAA	4	FA	50 mM NaPi pH 7.5, 150 mM NaCl and 10 mM beta-mercaptoehanol	25	66	1	40
CCCCGUAGAAAAUCUUAGU AAUCCUUCUUACAUUG	35	FA	50 mM NaPi pH 7.5, 150 mM NaCl and 10 mM beta-mercaptoehanol	25	0,023	0,0003	40

Supplementary Table S1: Summary of the binding affinity data published for Nrd1/Nab3 and Sen1 interactions with peptides or RNAs. Aminoacids in bold in the first column indicate phosphorylated residues. Abbreviations: FA (fluorescence Anisotropy), NMR (Nuclear Magnetic Resonance), ITC (Isothermal Titration

Calorimetry), KPi (Potassium phosphate buffer), NaPi (Sodium phosphate buffer) K_D (Apparent dissociation constant). References numbers as in main text

Supplementary data for
The Nrd1-Nab3-Sen1 transcription termination complex from a structural perspective

Belén Chaves-Arquero[1]* and José Manuel Pérez-Cañadillas[2]*

Protein	Domain	Residues	Technique	PDB code	Description
PCF11	CID	1-140	X-RAY	1SZ9	FREE PROTEIN
PCF11	CID	1-140	X-RAY	1SZA	CID-CTD Ser2p
PCF11	CID	1-138	X-RAY	2BF0	FREE PROTEIN
PCF11	zinc finger	538-608	NMR	1NAX	Structure of CCHC zinc finger domain of Pcf11
PCF11	Clp1 binding motif	454-563	X-RAY	2NPI	Clp1-ATP-Pcf11 complex
PCF11	Clp1 binding motif	454-563	X-RAY	4C0B	Clp1p-Pcf11p (454 -563) complex
PCF11	Clp1 binding motif	454-563	X-RAY	4C0H	Clp1p-Pcf11p (454 -563) complex
PCF11	Clp1 binding motif	454-563	X-RAY	4OI4	Clp1p-Pcf11p (454 -563) complex
PCF11	zinc finger	530-626	NMR	5M9Z	zinc-binding domains
RTT103	CID	3-131	NMR	2L0I	CID-CTD Ser2p
RTT103	CID	1-131	NMR	2KM4	CID
RTT103	homodimerization	1-131	NMR	5M48	homoDimerization domain
RTT103	CID	1-131	NMR	5LVF	CID-CTD thr4p
RTT103	CID	1-131	NMR	5M9D	CID-CTD Ser2p-Ser7p
RTT103	CID	5-131	NMR	5WOZ	CID
NRD1	CID	1-153	NMR	2LO6	CID-CTD Ser5p
NRD1	CID	1-153	NMR	2MOW	CID TRf4- NIM
NRD1	RBD	307-491	NMR		RBD Wrong
NRD1	CID	6-151	NMR	3CLJ	CID
NRD1	RBD	290-468	NMR	5O1T	RBD FREE
NRD1	RBD	301-489	X-RAY	5O1W	RBD FREE
NRD1	RBD	290-468	X-RAY	5O1X	RBD FREE
NRD1	RBD	290-468	X-RAY	5O1Y	RBD GUAA
NRD1	RBD	290-468	X-RAY	5O20	RBD UUAGUAAUC
NRD1	CID	1-153	NMR	6CG3	CID - Sen1 NIM
NRD1	CID	6-156	X-RAY	6O3W	CID-Sen1 NIM1
NRD1	CID	6-156	X-RAY	6O3X	CID-Sen1 NIM2
NRD1	CID	6-156	X-RAY	6O3Y	CID-Sen1 NIM3
NRD1	NAID	158-222	NMR	7PRD	chimera
NAB3	NRID	202-261	NMR	7PRD	chimera
NAB3	NRID	203-250	NMR	7PRE	free protien
NAB3	RRM	321-415	NMR	2KVI	free protein
NAB3	RRM	328-404	NMR	2L41	complex with UCUU
NAB3	RRM	329-404	X-RAY	2XNQ	free protein
NAB3	RRM	329-404	X-RAY	2XNR	Complex with UUCUUAUUCUUA
SEN1	HD	1095-1470, 1539-1904	X-RAY	5MZN	Helicase Domain
SEN1	HD	1095-1470, 1539-1904	X-RAY	6i59	Helicase Domain
SEN1	NIM	2052-2063	NMR	6CG3	CID - Sen1 NIM
SEN1	NIM	1882-1895	X-RAY	6O3W	CID-Sen1 NIM1

SEN1	NIM	2053-2064	X-RAY	6O3X	CID-Sen1 NIM2
SEN1	NIM	2181-2193	X-RAY	6O3Y	CID-Sen1 NIM3

Supplementary Table S2: Summary of experimental structural data available on the Protein Data Base (PDB).
